# Decoupling Effects of Electrostatic Gating on Electronic Transport and Interfacial Charge-Transfer Kinetics at Few-Layer Molybdenum Disulfide

**DOI:** 10.1021/acsnanoscienceau.2c00064

**Published:** 2023-02-20

**Authors:** Sonal Maroo, Yun Yu, Takashi Taniguchi, Kenji Watanabe, D. Kwabena Bediako

**Affiliations:** † Department of Chemistry, University of California, Berkeley, California 94720, United States; ^‡^International Center for Materials Nanoarchitectonics and ^§^Research Center for Functional Materials, National Institute for Materials Science, Tsukuba 305-0044, Japan; ∥ Chemical Sciences Division, Lawrence Berkeley National Laboratory, Berkeley, California 94720, United States

**Keywords:** SECCM, electrostatic gating, field-effect transistor, 2D MoS_2_, electrochemistry

## Abstract

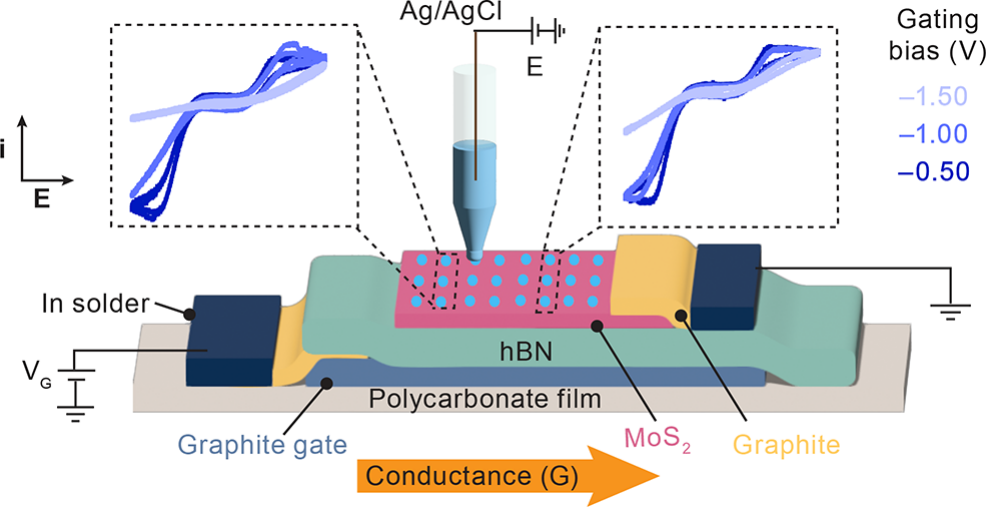

The electronic properties of electrode materials play a crucial role in defining their electrochemical behavior in energy conversion and storage devices. The assembly of van der Waals heterostructures and fabrication into mesoscopic devices enable the dependence of an electrochemical response on electronic properties to be systematically interrogated. Here, we evaluate the effect of charge carrier concentration on heterogeneous electron transfer at few-layer MoS_2_ electrodes by combining spatially resolved electrochemical measurements with field-effect electrostatic manipulation of band alignment. Steady-state cyclic voltammograms and finite-element simulations reveal a strong modulation of the measured electrochemical response for outer-sphere charge transfer at the electrostatic gate voltage. In addition, spatially resolved voltammetric responses, obtained at a series of locations at the surface of few-layer MoS_2_, reveal the governing role of in-plane charge transport on the electrochemical behavior of 2D electrodes, especially under conditions of low carrier densities.

Increasing societal energy demand requires the development of systems that efficiently interconvert electrical and chemical energy.^1^ Since electron transfer and electron transport constitute key steps in these interfacial processes,^2,3^ a deep understanding of the factors that underpin heterogeneous electron transfer and chemical reactivity is required. Marcus theory^4^ provides a powerful framework for understanding homogeneous outer-sphere electron-transfer reactions between two chemical species. Likewise, Gerischer’s formulation^5,6^ describes the heterogeneous ET rate constant, *k*_ET_, in the weak coupling (outer-sphere) limit. For a reduction reaction, *k*_ET_ is expressed as
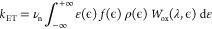
where ν_n_ is the nuclear frequency factor, ε(ϵ) is the proportionality function, *f*(ϵ) is the Fermi function, ρ(ϵ) is the density of states of the electrode, *W*_ox_(λ, ϵ) is the probability density function of the reactant (oxidized species, ox), and λ is the reorganization energy. Schmickler’s theory for electrocatalysis describes how electrochemical reactions in the strong-coupling (inner-sphere) limit, involving the adsorption of an intermediate at the electrode, are also significantly impacted by the structure and dispersion of the electronic bands in the electrode near the Fermi level, ϵ_F_.^7^ Indeed, several studies have reported the amplification of the interfacial charge-transfer processes caused by defects, edge sites, and grain boundaries on the electrode surface,^8−10^ which can be attributed to the localized enhancement in electronic states within their proximity to ϵ_F_. These theoretical and experimental studies underscore that interfacial reactivity may be strongly affected by modulating electronic structure. Field-effect transistors (FETs) are the essential components of contemporary electronics, serving as switches and amplifiers in a wide range of applications from smartphones and laptops to sensors and actuators.^11−13^ In these devices, an electric field applied via a gate electrode (i.e., electrostatic “gating”) is used to modulate the flow of current in the active semiconductor channels. For bulk semiconductors, applying an electric field perturbation alters the alignment of ϵ_F_ with the conduction/valence bands (and consequently the charge-carrier density) at the semiconductor–dielectric interface but not in the bulk of the material, producing the well-known “band bending” effect.^14^ In low-dimensional semiconductors, including two-dimensional (2D) layers, electrostatic gating controls the band alignments and densities of charge carriers throughout the material.^15−17^ 2*H*-Molybdenum disulfide is a van der Waals (vdW) layered semiconductor with an electronic band gap of 1.29 eV for bulk crystals and 1.8 eV in the monolayer limit.^18−20^ Applying an electric field destabilizes the electronic bands with respect to ϵ_F_, thereby altering the density of electronic states at ϵ_F_ as depicted in Figure 1a. The electronic property manipulation by this FET approach is highly controllable and therefore can provide a powerful means of systematically studying the influence of the density of electronic states on interfacial charge transfer kinetics that may be explained in the framework of the Marcus–Gerischer model; the applied electrostatic gate controls the alignment of the band edges with respect to ϵ_F_, and the electrochemical polarization generally controls the alignment of ϵ_F_ with respect to the solution redox couple (Figure 1b), although we note that the electrochemical polarization can also further gate the semiconductor.^21,22^

**Figure 1:**
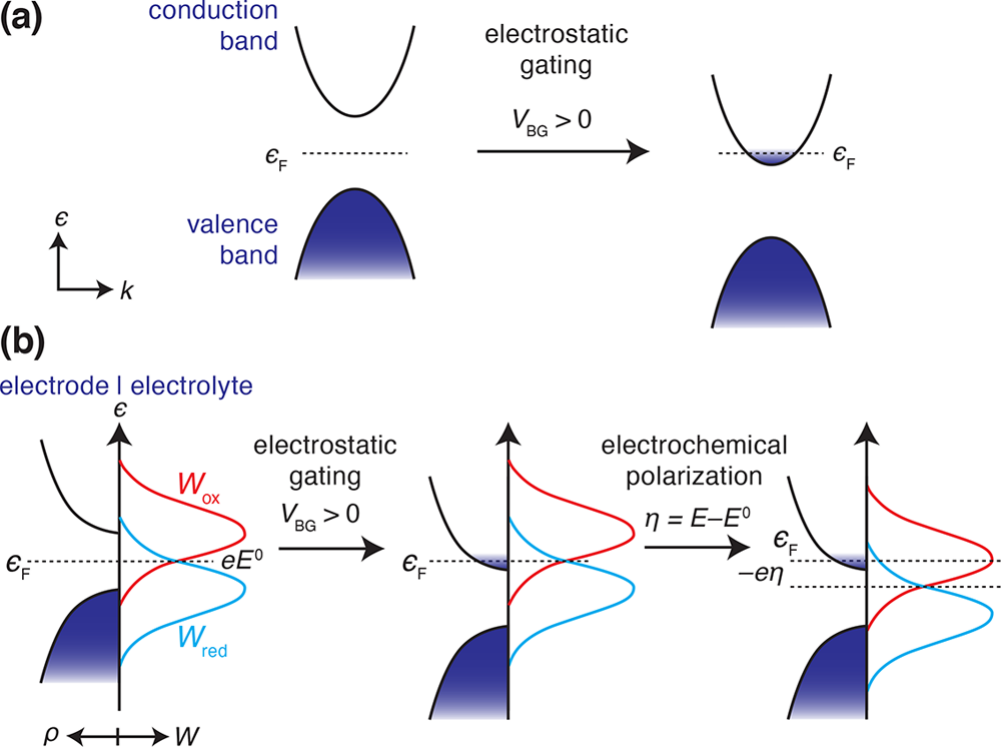
(a) Illustration of the shift in the band edge positions of a semiconducting material relative to ϵ_F_ upon applying an electrostatic gate voltage, *V*_BG_. (b) “Gerischer” schematic illustrating ϵ_F_ of the semiconducting electrode in relation to the probability distributions of occupied (*W*_red_) and empty (*W*_ox_) states in solution at equilibrium and after applying an electrostatic gate voltage, *V*_BG_, followed by a cathodic overpotential, η.

Along these lines, previous studies have demonstrated that the heterogeneous charge-transfer kinetics at MoS_2_ monolayers can be strongly modulated by applying an external electric field on the working electrodes,^23,24^ allowing charge-transfer kinetics at monolayer MoS_2_ electrodes to be continuously and reversibly tuned from irreversible to nearly reversible (controlling the standard rate constant over 100-fold) with an applied bottom-gate bias (*V*_BG_). Changes in electronic conductivity with electrostatic gating^13,22,25^ have also been implicated in affecting the interfacial reactivity. The surface conductance of MoS_2_ crystals has been strongly correlated with electrocatalytic activity in electrochemical systems via a “self-gating” effect of the electrochemical polarization itself that produces a highly conductive surface.^22,25^ Together, these studies suggest that in an electrochemical system involving semiconducting electrodes, electrostatic gating (whether by the electrolyte itself or a solid-state gate) can affect both intrinsic interfacial electrokinetic behavior (as described formally by the Marcus–Gerischer equation) and in-plane electronic transport (conductivity), resulting in the net electrochemical response of the system. However, these effects have yet to be deconvoluted in a single set of experiments.

Here we decouple the effects of in-plane charge transport and intrinsic electrokinetics by employing the scanning electrochemical cell microscopy (SECCM) technique^26,27^ in an FET configuration. Our FET-SECCM approach enables the acquisition of electrochemical measurements exclusively in select nanoscale regions of the MoS_2_ basal plane while keeping the remainder of the flake dry and isolated from concomitant electrostatic gating and conductivity changes by the electrochemical polarization itself; instead, the conductivity of the flake is controlled by the separate electrostatic gate. We obtain spatially resolved voltammetric responses at a series of locations at the surface of few-layer MoS_2_ as a function of the gating voltage. Our results reveal the crucial role of in-plane charge transport in governing the electrochemical responses of 2D semiconducting electrodes, especially under conditions of low carrier densities. The experimental approach and results presented here also emphasize the versatility of 2D materials and vdW heterostructures as platforms for probing the physicochemical principles that underpin interfacial electron-transfer reactions.

The atomically thin MoS_2_, graphene, and boron nitride (hBN) flakes used in this work were mechanically exfoliated onto SiO_2_ (285 nm)/Si substrates from their bulk crystals using the Scotch tape method.^28,29^ Flake thicknesses were evaluated using optical contrast (SI Figure 1)^30,31^ and atomic force microscopy, AFM (SI Figure 2). The thicknesses of MoS_2_ flakes were verified using confocal Raman spectroscopy and photoluminescence spectroscopy (SI Figure 3). Specifically, monolayers of MoS_2_ were identified by their strong photoluminescence that arises from a direct band gap in the monolayer limit.^18,32,33^ In all electrochemical measurements, the MoS_2_ electrodes were fabricated in a field-effect transistor (FET) configuration, as depicted in Figure 2a. Graphite, hBN, and MoS_2_ flakes were sequentially stacked over each other by using the vdW dry-transfer method.^34^ Further details on sample preparation are provided in the Supporting Information. MoS_2_ flakes with thicknesses of one to three layers were assembled on top of an hBN crystal (20–50 nm) as an atomically flat dielectric and a graphite flake as the bottom gate electrode. Figure 2b shows an optical micrograph of a typical vdW heterostructure MoS_2_-based FET device used in this work.

**Figure 2:**
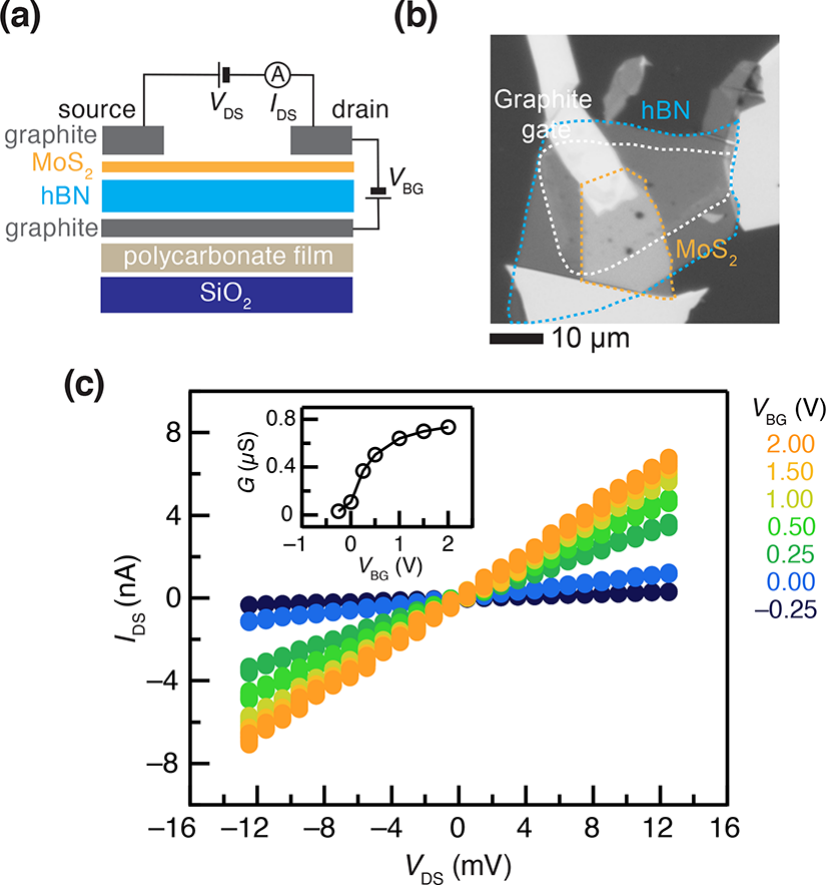
(a) Schematic of the FET device used for electrochemical measurements in this work. *V*_DS_: drain–source potential. *V*_BG_: bottom-gate voltage. (b) Optical micrograph of a representative bottom-gated monolayer MoS_2_ FET device. (c) Two-probe *I*_DS_ vs *V*_DS_ curves as a function of *V*_BG_ at a monolayer MoS_2_. The inset shows the two-probe conductance, *G*, measured as *I*_DS_/*V*_DS_.

The voltage (*V*_BG_) applied to the graphite bottom gate was used to alter the carrier density and exert control over the band alignment. Specifically, the position of ϵ_F_ shifts either toward or away from the conduction band edge when *V*_BG_ is modulated as illustrated in Figure 1a. Figure 2c shows the source-to-drain current profile obtained for different *V*_BG_ values from −0.25 to +2.0 V. The observation of substantial *I*_DS_ at *V*_BG_ = 0 V implies that our MoS_2_ flakes used in these devices are n-doped possibly due to S vacancies, as is typically the case in transition-metal dichalcogenides.^35^ This assertion is further confirmed by the A- and B-exciton peak intensity ratios obtained from the PL spectra (SI Figure 3b). Figure 2c shows that, as expected, the electrical conductance, *G*, of the MoS_2_ layers in these devices (*G* = *I*_DS_/*V*_DS_) increases with *V*_BG_, corresponding to electron accumulation at MoS_2_. *G* saturates at higher *V*_BG_ as ϵ_F_ resides well within the conduction band (Figure 2c inset).

In an electrochemical measurement for such an FET device, we would expect that in addition to tuning the conductance, the position of ϵ_F_ controlled by *V*_BG_ would also affect the extent of overlap between filled electronic states of the semiconductor electrode and the probability distribution functions of redox molecules residing in an electrolyte in contact with the semiconductor (Figure 1b). Therefore, in an electrochemical measurement, we would also expect *V*_BG_ to modulate the intrinsic interfacial electron-transfer kinetics. To probe this effect, we performed SECCM measurements of MoS_2_-based devices in the FET configuration. SECCM is a scanning probe technique that enables the interrogation of electron-transfer reactions at the nanoscale using an electrolyte-filled nanopipette that is positioned/scanned over the sample, allowing a micro/nanoelectrochemical cell to be established by contact of the electrolyte meniscus at the base of the pipette and the sample surface.^26,27^ As depicted in Figure 3a, we employed quartz nanopipette probes of diameter ∼500 nm (SI Figure 4) that were filled with an aqueous electrolyte of 1 mM hexaammineruthenium(III) chloride and 100 mM potassium chloride to make meniscus contact with the gate-tunable MoS_2_ surface, creating an enclosed electrochemical cell in which localized voltammetry is performed for a series of *V*_BG_ values. (See the Supporting Information for additional details of the measurement.) The radii and taper angles of nanopipettes were determined from transmission electron micrographs (SI Figure 4). Figure 3b shows an optical micrograph of a monolayer MoS_2_ device measured in this manner, using an hBN flake of 20 nm thickness as the gate dielectric.

**Figure 3:**
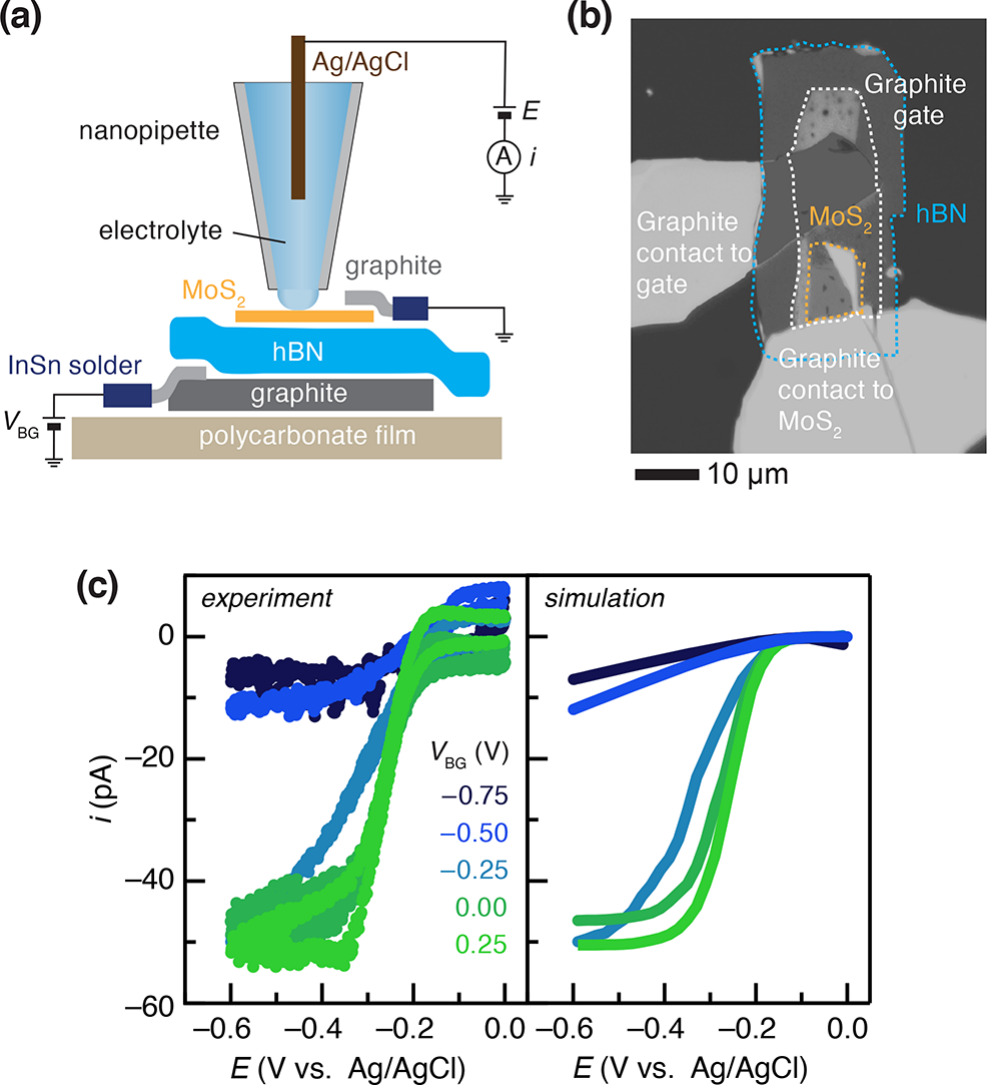
(a) Schematic of a local voltammetric measurement in the SECCM setup. (b) Optical micrograph of a bottom-gated monolayer MoS_2_ electrode (hBN thickness: 20 nm). (c) Left: experimental cyclic voltammograms of 1 mM Ru(NH_3_)_6_^3+^ in 0.1 M KCl solution as a function of *V*_BG_. Scan rate: 200 mV/s. Right: simulated voltammograms using parameters in Table 1.

Experimental SECCM cyclic voltammograms of Ru(NH_3_)_6_^3+^ reduction obtained with nanopipettes probing the center of the gated monolayer MoS_2_ flake are shown on the left of Figure 3c. Initially, even at *V*_BG_ = 0 V, the cyclic voltammogram of Ru(NH_3_)_6_^3+^ exhibits nearly reversible characteristics, which are attributed to the pre-existing n-doping of MoS_2_ discussed above. As *V*_BG_ is increased to 0.25 V, signifying further electron doping, the reaction shows a measurable enhancement in the apparent interfacial kinetics, displaying a fully electrochemically reversible response, with the plateau (diffusion-limited) current density equaling that of bulk graphite (see SI Figure 5). As *V*_BG_ is varied from 0.25 to −0.75 V, which involves p-doping (or equivalently, a reduction in n-doping), we observe an anodic shift of the oxidation wave and a decrease in the plateau current in each voltammogram (green to blue). A similar response was observed in the case of the 1 mM ferrocene methanol redox couple (SI Figure 5a). This behavior can be ascribed to an upward shift in the conduction band edge position relative to ϵ_F_. This shift of ϵ_F_ toward the band gap necessarily reduces the charge-carrier concentration, which would substantially impact the electronic density of states available to mediate the interfacial electron transfer as well as the energy state overlap integral between empty (occupied) states of Ru(NH_3_)_6_^3+(2+)^ and an occupied (empty) state of the same energy in the MoS_2_ electrode. These factors would relate to the intrinsic electrokinetic behavior of the material. However, changes in the conductance of MoS_2_ should also be expected.

To understand the contributions of intrinsic electrokinetics as well as electronic transport to the electrochemical responses, we used finite-element simulations (COMSOL Multiphysics v.5.6)^36^ to model the voltammetric responses and estimate electrochemical rate constants (*k*^0^) and conductance values (*G*) in our system (SI Figures 6–8). The axisymmetric geometry of the electrochemical cell was modeled with a cylindrical meniscus and a nanopipette, with dimensions determined from TEM images and the limiting current density. The chemical and electron-transfer kinetics of the redox couples Ru(NH_3_)_6_^+3/+2^ and FcMeOH^3+/2+^ were modeled using the Nernst–Planck model and Butler–Volmer equations. The in-plane resistance of the electrode was taken into account by modeling it as a resistive film. The initial guesses of *G* were informed from the measurements described in Figure 2c, after which *k*^0^ and *G* were then iteratively optimized. The simulated voltammograms were compared to experimental data to refine the simulation parameters. Further details on the finite-element simulation parameters and the procedures for estimating kinetic and transport parameters can be found in the Supporting Information. Table 1 details the resultant dependence of *k*^0^ and *G* on *V*_BG_ for Ru(NH_3_)_6_^+3/+2^. For comparisons among devices, we also compute and tabulate the electric field  = *V*_BG_/*d*_hBN_, where *d*_hBN_ is the thickness of the hBN dielectric used in device fabrication (determined from AFM, SI Figure 2), which typically varies from device to device. These data suggest that the response of the system to electrostatic gating involves the intertwined effects of changing conductivity, and hence conductance, and the intrinsic interfacial kinetics, owing to modulation in electronic densities of states as well as the overlap between the energy states of the reactant and electrode.

**Table 1:** Values of *G* and *k*^0^ for Ru(NH_3_)_6_^+3/+2^ from the Simulation of CVs at Monolayer MoS_2_

*V*_BG_ (V)	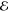 (mV/nm)	*k*^0^ (cm/s)	*G* (nS)
0.25	12.5	0.6	≥2.5
0.0	0	0.4	≥2.0
–0.25	–12.5	0.2	0.43
–0.50	–25.0	0.02	0.09
–0.75	–37.5	0.004	0.05

These insights notwithstanding, a stronger segregation of the contributions of interfacial kinetics and electronic transport on the electrochemical response required a measurement scheme that provided greater orthogonality in the manipulation of *G* and *k*^0^. To gain a deeper understanding of the independent impact of *G* on the overall electrochemical response, we acquired spatially resolved voltammetric responses by positioning nanopipettes filled with 2.0 mM hexaammineruthenium(III) chloride, 1.0 mM ferrocene methanol, and 100 mM aqueous potassium chloride at a series of locations at the surface of a bottom-gated trilayer MoS_2_ electrode (Figure 4a). We note that
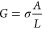
where σ is the conductivity, *L* is the length of the channel away from the electrical contact, and *A* is the cross-sectional area of the channel. We can then define a surface conductivity, σ′, as σ′ = *σA*. Accordingly, for a fixed *V*_BG_, σ′ would remain constant, yet as we move the pipette from one position to another, *G* would change as *L* is varied. Figure 4b shows cyclic voltammograms of ferrocene methanol and hexaammineruthenium(III/II) obtained at a series of points at the MoS_2_ surface with changing distances of 1.4, 9.4, and 16.6 μm from the point of SECCM measurement to the terminal graphite contact.

**Figure 4:**
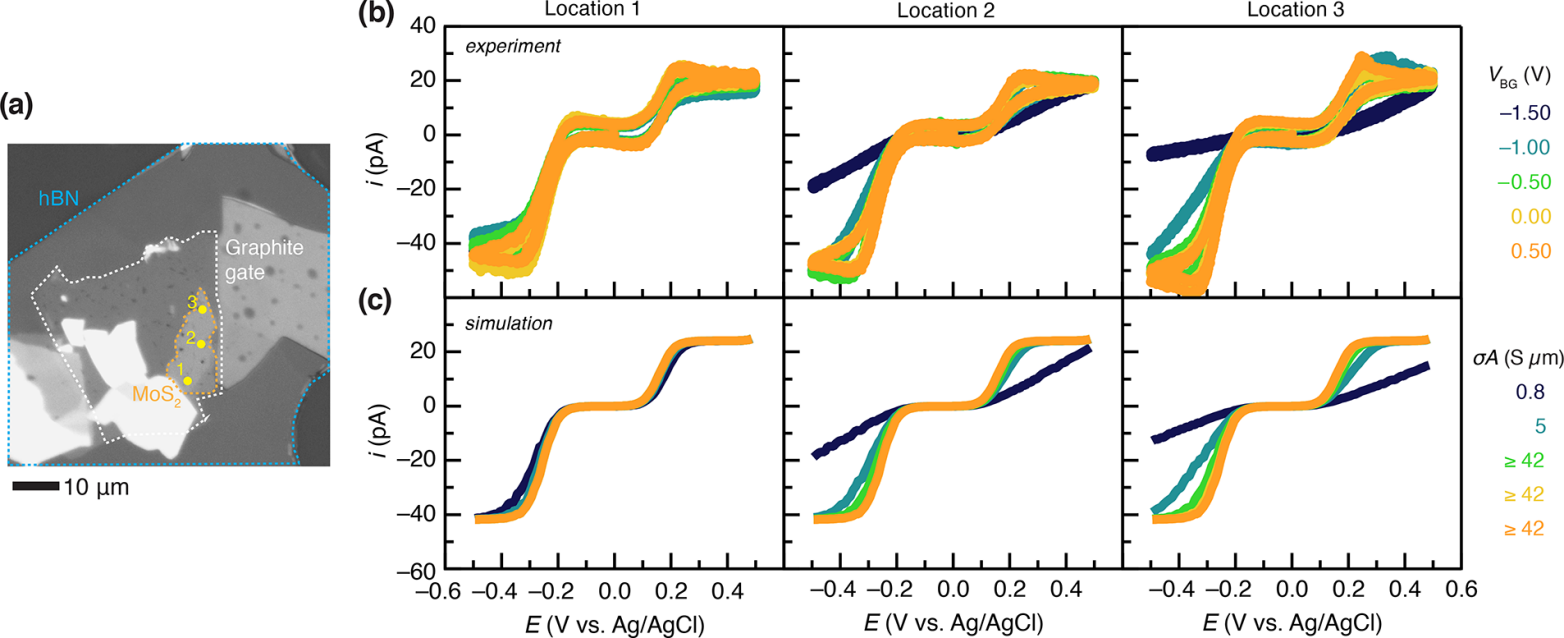
(a) Optical image of a gated three-layer MoS_2_ electrode using an hBN dielectric of thickness 40 nm. Marked spots 1–3 represent the locations probed by SECCM, varying the distance, *L*, from measurement to the graphite electric contact. (b) Cyclic voltammograms of 1 mM FcMeOH and 2 mM Ru(NH_3_)_6_^3+^ in 0.1 M KCl solution as a function of *V*_BG_ at locations 1 (left), 2 (middle), and 3 (right). Scan rate = 200 mV/s. (c) Simulated cyclic voltammograms (scan rate = 200 mV/s) using the values of *k*^0^ and *G* are detailed in Table 2. The effects of *V*_BG_ are incorporated via a changing surface conductivity, *σ'* = *σA*.

At *V*_BG_ = 0 V, voltammograms of FcMeOH and Ru(NH_3_)_6_^3+^ exhibit electrochemically reversible responses at all three locations, consistent with an MoS_2_ surface that is natively n-doped with sufficient charge carriers to mediate the heterogeneous electron-transfer reactions and also conduct electrical charge without a substantial ohmic drop across the flake. As *V*_BG_ was varied toward −1.5 V, hole doping via the electric field effect caused pronounced differences in the electrochemical responses at different locations at the MoS_2_ surface. The voltammogram at location 1, 1.4 μm away from the graphite contact, remained largely unaffected, exhibiting electrochemical reversibility, while the voltammogram at location 3, ∼17 μm away from the graphite contact, displayed a substantial decrease in the Faradaic current associated with both FcMeOH and Ru(NH_3_)_6_^3+^, producing ostensibly irreversible responses by *V*_BG_ = −1.50 V.

The set of measurements displayed in Figure 4b provides a means of systematically probing the effects of electrostatic gating on conductivity and intrinsic charge-transfer kinetics by iterative comparison with finite-element simulations. In simulations, the effect of electrostatic gating was incorporated through a *V*_BG_-dependent but location-independent set of *k*^0^ and σ′ values while further considering changes to the in-plane electronic transport via a location-dependent (inversely proportional to *L*) set of *G* values at each site. CV simulations within this framework (SI Figures 8 and 9) are instructive, revealing that at low conductance levels (*G* ≤ 0.05 nS) increasing *k*^0^ even by 2 orders of magnitude does not substantially influence the current profile. Instead, *G* overwhelmingly governs the rate of interfacial electron transfer in this regime. However, as *G* increases, both *G* and *k*^0^ contribute substantially to the overall electrochemical behavior. SI Figures 8 and 9 show that ultimately for *G* ≥ 0.5 nS increasing *G* further does not influence the current profile and *k*^0^ solely determines the overall interfacial electron-transfer rate.

Against this backdrop, we iteratively optimized *k*^0^ and *G* to reproduce the experimental data in Figure 4b. The simulation parameters that were found to most closely replicate the experimental data are detailed in Table 2, and the simulated voltammograms are presented in Figure 4c. We find good replication of the redox behavior for both FcMeOH and Ru(NH_3_)_6_^3+/2+^ using effectively identical *k*^0^ values, which are the singular *k*^0^ values presented in Table 2. We note that for the reasons described above, in some locations and at some values of *V*_BG_, we can rigorously provide only lower-limit values of *k*^0^ and/or *G*. Nevertheless, these results provide an instructive illustration of the interplay between in-plane charge transport and intrinsic interfacial electrokinetics.

**Table 2:** Location-Dependent Values of *G* and *k*^0^ for Electrochemical Responses of FcMeOH and Ru(NH_3_)_6_^3+/2+^ at Trilayer MoS_2_

			*G* (nS)	*G* (nS)	*G* (nS)
*V*_BG_ (V)	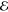 (mV/nm)	*k*^0^ (cm/s)	at *L* = 1.4 μm	at *L* = 9.4 μm	at *L* = 17 μm
0.5	12.5	≥0.1	≥2.5	≥2.5	≥2.5
0	0	≥0.1	≥2.5	≥2.5	≥2.5
–0.5	–12.5	≥0.1	≥2.5	≥2.5	≥2.5
–1.0	–25.0	0.085 ± 0.005	≥2.5	0.55 ± 0.06	0.290 ± 0.003
–1.5	–37.5	0.055 ± 0.002	≥2.5	0.083 ± 0.003	0.047 ± 0.004

In conclusion, electrostatic gating of semiconducting electrodes results in the modulation of intrinsic electrochemical kinetics as well as the electronic transport properties. The FET scheme provides an orthogonal knob to control interfacial charge transfer, distinct from the applied electrochemical bias, but the effects of in-plane transport must be considered. This work demonstrates that vdW heterostructures of few-layer MoS_2_, graphite, and hBN provide a distinctive platform for interrogating charge-transfer kinetics and electronic transport effects when coupled with SECCM. SECCM enables the exclusion of electrochemical gating effects on in-plane transport, allowing electronic transport to be controlled exclusively by the solid-state gate. Combined experimental measurements and finite-element simulations show how in-plane charge transport has a pronounced effect on the apparent electron-transfer kinetics at semiconducting electrodes, especially at lower charge-carrier densities.

## Supporting Information Available

General methods, additional experimental data, and finite element simulations (PDF)
