# Supplementary material for: Decoupling Effects of Electrostatic Gating on Electronic Transport and Interfacial Charge-Transfer Kinetics at Few-Layer Molybdenum Disulfide
Source: ACS Nanosci Au. 2023 Feb 20;3(3):204–10. doi: 10.1021/acsnanoscienceau.2c00064 (PMC10288603; doi:10.1021/acsnanoscienceau.2c00064)
Supplement: Supplementary file 1 — ng2c00064_si_001.pdf [file ng2c00064_si_001.pdf]

Supplementary Information for

Decoupling effects of electrostatic gating on electronic transport and interfacial charge transfer kinetics at few-layer molybdenum disulfide

Sonal Maroo[1], Yun Yu[1,†], Takashi Taniguchi[2], Kenji Watanabe[3], and D. Kwabena Bediako[1,4,*]

[1] *Department of Chemistry, University of California, Berkeley, CA 94720, USA*
[2] *International Center for Materials Nanoarchitectonics, National Institute for Materials Science, Tsukuba, Japan*
[3] *Research Center for Functional Materials, National Institute for Materials Science, Tsukuba, Japan*
[4] *Chemical Sciences Division, Lawrence Berkeley National Laboratory, Berkeley, CA 94720, USA*
[†] *Current Address: Department of Chemistry, George Mason University, Fairfax, VA 22030, USA*
[*] *Correspondence to: bediako@berkeley.edu*

1 Sample preparation

Graphene (Graphene Supermarket) and hBN (used as received from T. Taniguchi and K. Watanabe) flakes were mechanically exfoliated outside the glovebox using adhesive tape (Scotch Magic tape). The mechanical exfoliation of 2H-MoS_2 (HQ graphene) was performed in an Ar-filled glovebox using adhesive blue tape (Ultron Systems, Inc.). For thin samples (< 6 layers) of MoS_2, previous studies[1,2] suggest that the thickness can be reliably identified using optical contrast (C) measurement which is defined as:

$$C = \frac{I_{substrate} - I_{sample}}{I_{substrate} + I_{sample}}$$

The intensities of the few-layer MoS_2 and substrate obtained from the brightness profile of the red channel of the optical micrographs were used to calculate C. C was found to be 0.12 ± 0.02 for monolayer MoS_2 and 0.27 ± 0.02 for trilayer MoS_2, consistent with previous studies.[1,2] The MoS_2 flakes were fabricated into devices in a field effect transistor (FET) configuration (Fig. 2a) with a solid-state graphite bottom gate using the dry transfer method.[3] A polymeric stamp was prepared by placing a thin 2 mm × 2mm film of poly(bisphenol A carbonate), PC, on a 4 mm × 4 mm polydimethylsiloxane (PDMS) stamp attached to a glass

slide. The PC/PDMS polymer stamp was used to pick up a thin graphite flake (5–6 layers) for use as the bottom gate using a temperature-controlled heating stage (Instec) equipped with an optical microscope (Mitutoyo FS70) and a micro-manipulation stage (MP-285, Sutter Instrument). Thicker graphite flakes (5 – 50 nm) were sequentially picked up to make contact with the graphite gate. Subsequently, a thin hBN crystal (20 – 50 nm) was picked up by the graphite gate followed by the MoS_2 flake. Finally, another set of thin graphite flakes was stacked in contact with the MoS_2. The entire stamp with vdW heterostructure attached was then transferred to a glass slide with the MoS_2 side facing upwards. InSn solder was then used to make electrical contact to the graphite flakes as described previously.[4]

2 Raman and PL measurements

The thickness of MoS_2 flakes was verified using Raman and photoluminescence (PL) spectroscopy (Horiba Multiline LabRam Evolution) using a 532 nm laser excitation, dispersed by an 1800 g/mm grating, 1% ND filter with 3 s acquisition time and 3 accumulations. Baseline correction was performed by fitting a polynomial curve to the background. The Raman spectra (SI Fig. 3b) of the MoS_2 samples display the in-plane E^1_{2g} mode at 385 cm^{-1} and out-of-plane A_{1g} mode at 404 cm^{-1}, consistent with previous data for monolayer MoS_2.[5] The PL spectra (SI Fig. 3c) acquired at the same position exhibit peaks at about 620 nm and 670 nm corresponding to the A- and B- excitonic transitions.[6] Since A- and B-exciton photoluminescence intensity ratio depicts the doping level for MoS_2 monolayers, a smaller ratio, in this case, implied that our flakes were doped.[6,7]

3 SECCM experiment

We use the general approach to SECCM measurements that has been described previously.[4,8,9] SECCM measurements were performed on a home-built instrument. Quartz nanopipettes approximately 500 nm in diameter were filled with electrolyte and an Ag/AgCl quasi-reference counter electrode (QRCE) was inserted. A potential was then applied between the working electrode (the MoS_2-based FET device) and the QRCE. The probe was made to slowly approach the working electrode. Once the electrolyte droplet made meniscus

contact with the electrode, an electrochemical current was detected due to the potential difference between the electrode and QRCE, which is also used for feedback. This signal terminated further approach to the surface. The electrochemical cell formed by the meniscus of the pipette was then used to carry out the measurements described in the manuscript. The probe could then be withdrawn and moved to a new position on the surface for subsequent measurements.

4 Finite-element simulations

The finite-element method was employed using COMSOL Multiphysics (version 5.6)[10] to simulate the cyclic voltammetry responses in the SECCM experiments. The "electroanalysis" module was utilized to simulate the mass transport of redox species in the electrochemical cell defined by the nanopipette and the meniscus. The 2-D axisymmetric geometry of the electrochemical cell was built with $r = 0$ as the symmetry axis (SI Fig. 6). The meniscus formed between the nanopipette and the sample surface was simulated as a cylinder with a height, h. It is important to note that the contact angle of few-layer MoS_2 can exhibit a range of values between 70° to 90°.[11] To evaluate the effect of this variability, simulations were performed to determine the percentage deviation in the k^0 values for the extreme cases of $\theta = 70°$ and 90°. The simulations showed a maximum deviation of 20 % in the k^0 values. However, it should be noted that this deviation affects all data points and the relative variations in these values primarily result from the shift in Fermi level caused by electrostatic gating. In addition, $l = 30$ μm was set to ensure that the simulation space is much larger than the pipette size. The pipette radius, a_s, and the taper angle, θ_P, were determined from TEM images of the nanopipette (SI Fig. 4). A survey of multiple nanopipettes prepared using identical parameters (SI Fig. 4) showed that the cone angles are highly consistent (14.1 ± 0.3°) but there is a modest distribution of aperture sizes (420 – 550 nm). COMSOL simulations for $Ru(NH_3)_6^{3+/2+}$obtained using $a_s = 250$ nm and an electrochemically reversible condition ($k^0 \gg 1$ cm/s) showed that the taper angle has a negligible impact on the current profile for that range of variation. However, for a fixed taper angle ($\tan\theta = 0.25$), the limiting current density changes upon varying a_s. Hence, the

limiting current density was used as an additional parameter for fitting experimental data (*i.e.* to estimate a_s). Previous experiments showed that mass transport is weakly affected by the meniscus height, h, and therefore was set the same as a_s.[4,10]

The Nernst–Planck equation was employed to study the chemical transport of the reactant and product species of the redox couple:

$$\mathbf{N_i} = -D_i \nabla c_i - z_i \mu_i F c_i \nabla \phi_l + c_i \mathbf{u}$$

Here, N_i is the flux of species i, D_i is the diffusion coefficient, c_i is the concentration, z_i is the charge number, μ_i is the mobility, ϕ_l is the electrolyte potential and u is the velocity field of the solvent. For studies of the $Ru(NH_3)_6^{3+/2+}$ couple, D_O and D_R were set to 6.5×10^{-6} cm^2/s.[12] $\alpha = 0.5$ and E^0 = –0.25 V vs. Ag/AgCl were used as constant values for simulation which is consistent with previous studies.[13] For studies of the $FcMeOH^{3+/2+}$ couple, $D_O = D_R = 7.6 \times 10^{-6} cm^2/s$, $\alpha = 1.0$ and $E^0 = 0.15$ V vs. Ag/AgCl were used for simulation.[4,14] We also added 0.1 M KCl which acts as a supporting electrolyte to increase the conductivity of the electrolyte without interfering with the reaction chemistry. Under these conditions, the resistance of the solution is sufficiently low that the electric field is negligible, and it is reasonable to assume that $\phi_l = 0$. In the absence of convection ($u = 0$), the equation reduces to Fick's first law of diffusion:

$$\mathbf{N_i} = -D_i \nabla c_i$$

After considering the mass balance and replacing N_i with current density $\mathbf{J_i}(\mathbf{J_i} = -D_i \nabla c_i)$, we get following steady-state mass transport equation:

$$\frac{\partial c}{\partial t} + \nabla \cdot \mathbf{J_i} = R_i$$

R_i represents a source or sink term, typically due to a chemical reaction (oxidation or re-

duction in our case). We used the Butler–Volmer equation to describe the heterogeneous electron-transfer kinetics:

$$i_{loc} = nFk_O \left(c_A exp\left(\frac{(n-\alpha_c)F\eta}{RT}\right) - c_B exp\left(\frac{-\alpha_c F\eta}{RT}\right)\right)$$

Here, k^0 is the heterogeneous rate constant of the reaction, α_c is the cathodic transfer coefficient, η is the overpotential at the working electrode, and c_A and c_B are the bulk concentrations of reduced and oxidized species, respectively. The following equations were employed to calculate the total current density:

$$\mathbf{n} \cdot \mathbf{i_l} = i_{total}$$

Here, i_l denotes the current density vector in the electrolyte and n is the normal vector of the boundary pointing into the domain. The charge transfer reactions at the interface between an electrode and an electrolyte domain generate a normal current flux (i_{total}) that equals the sum of all reaction currents:

$$i_{total} = \sum_m i_{loc,m} + i_{dl}$$

Here, $i_{loc,m}$ denotes the local charge transfer current density for reaction 'm'. The film resistance feature under the 'electrode surface' node in COMSOL was used to include an additional potential drop to account for the in-plane ohmic resistance for our spatially resolved studies and also to account for the change in conductivity due to the applied bottom gate voltage. MoS_2 was modeled as a resistive film of the electrode and the thickness of the bulk of the electrode was set to zero. The potential drop over the film $\Delta\Phi_{s,film}$ was introduced as:

$$\Delta\Phi_{s,film} = R_{film} i_{total} \qquad R_{film} = A/G$$

Here, R_{film} is the surface resistance (SI unit: Ωm^2), A is the area of the electrode surface and G is the conductance of the electrode surface.[10] A series of simulated voltammograms with varying k^0 and G values were obtained and compared to the experimental data (for

both forward and reverse waves). The best match was determined by replicating the limiting current and the CV midpoint values, and then a least mean square error analysis was employed to refine the simulation parameters.

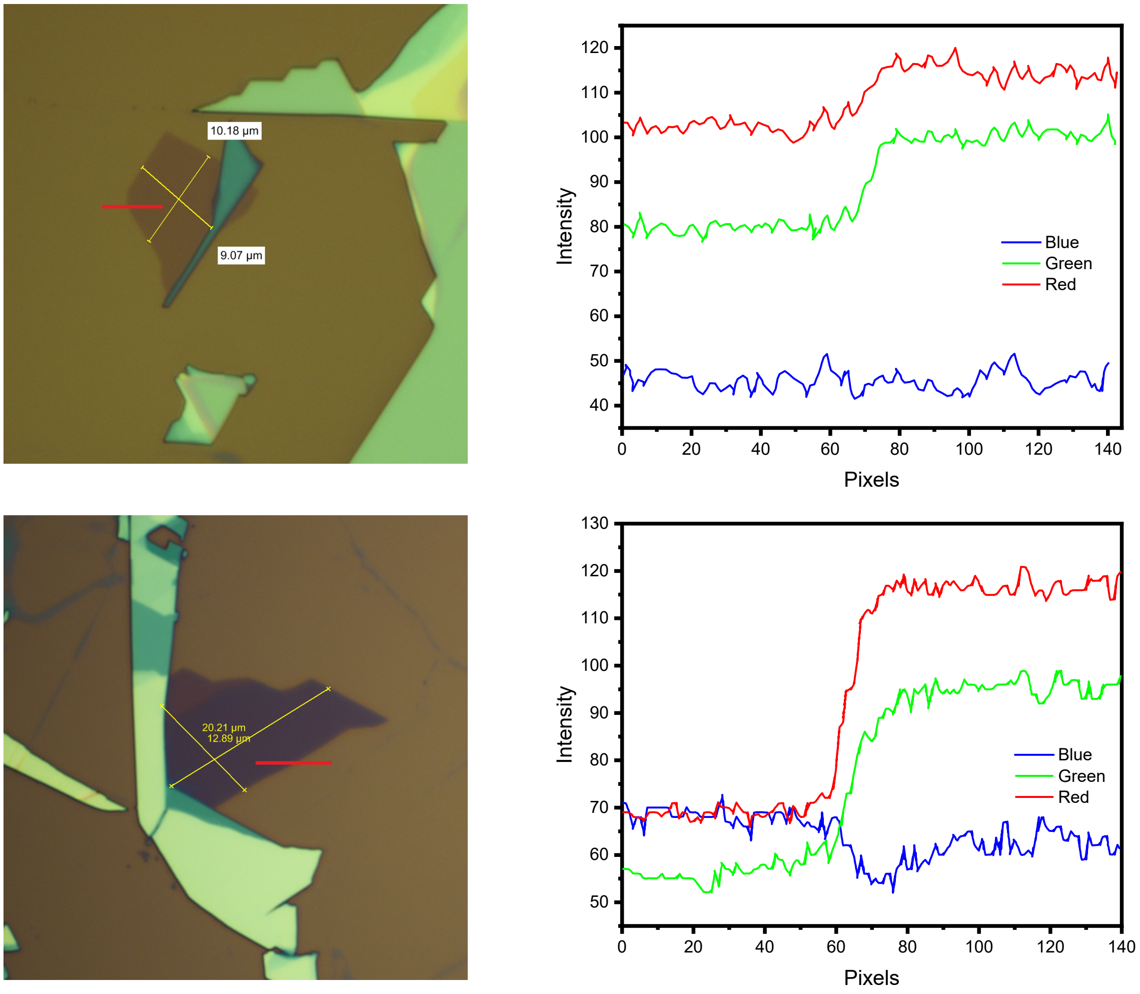

SI Fig. 1: Left – Optical image of the MoS_2 flakes employed in the experiment deposited on 285 nm SiO_2/Si. Right – The intensity profile along the red line for blue, green and red channels.

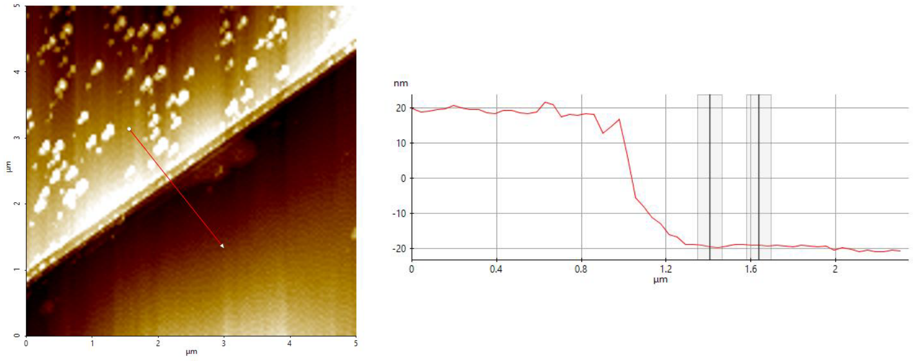

SI Fig. 2: AFM height image of hBN flake on 285 nm SiO_2/Si used in trilayer MoS_2 based FET experiment

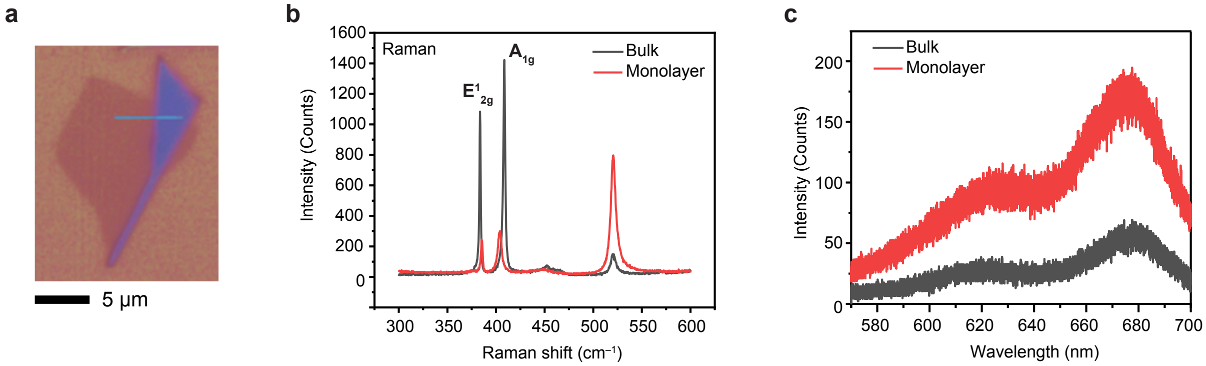

SI Fig. 3: (**a**), Optical image of monolayer MoS_2 flake. (**b**), Raman spectra and (**c**), photoluminescence spectra collected at different points (the blue line in (a)) of the unstacked MoS_2 flake.

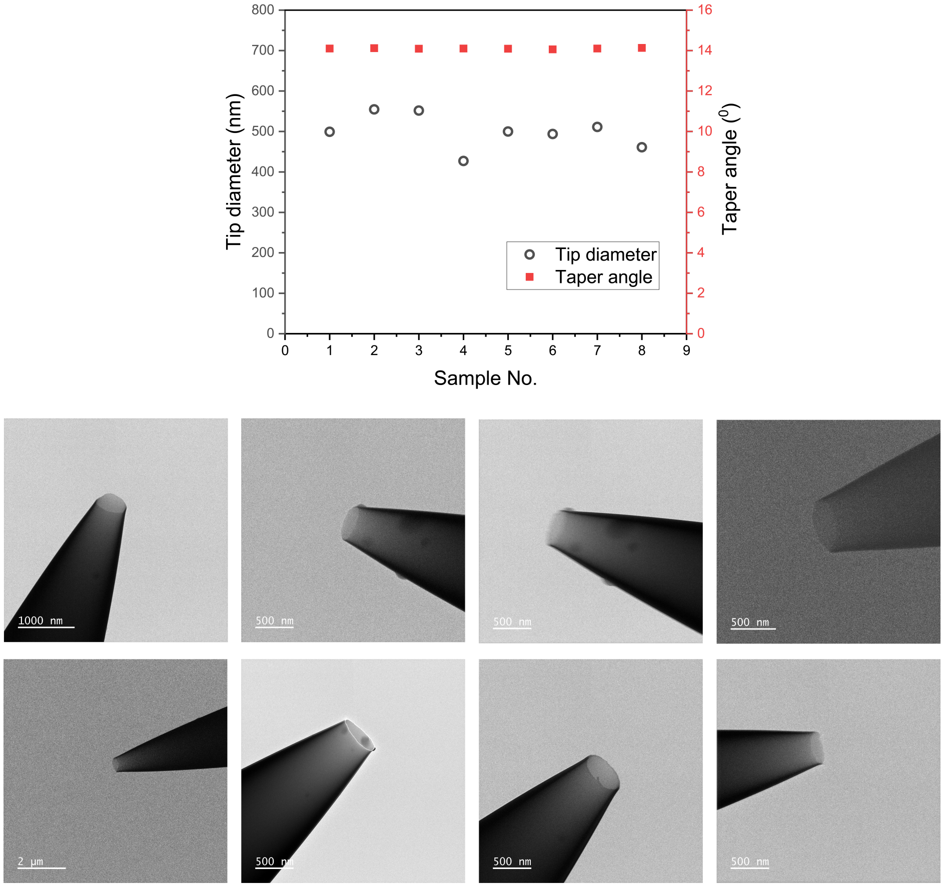

SI Fig. 4: Survey of nanopipette geometries showing the taper angle (14.1±0.3°) and the orifice diameter (430 – 570 nm) obtained from the TEM images of 8 nanopipettes.

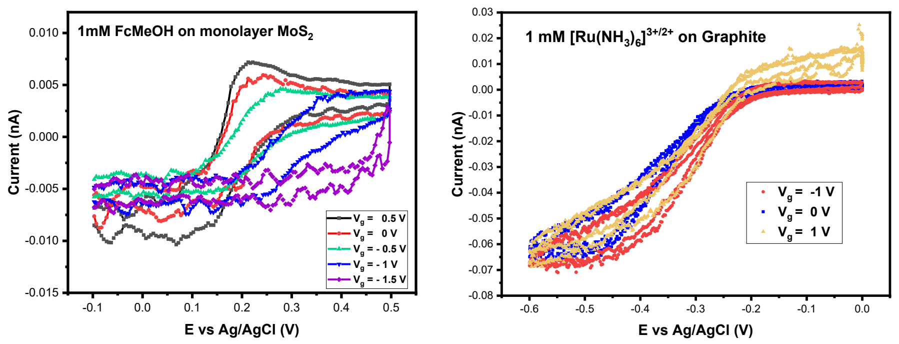

SI Fig. 5: Cyclic voltammograms of– (**a**), 1 mM FcMeOH in 0.1 M KCl solution on monolayer MoS_2 and (**b**), Cyclic voltammograms of 1 mM $Ru(NH_3)_6^{3+/2+}$ in 0.1 M KCl solution on bulk graphite, as a function of V_{BG}. Scan rate = 200 mV/s

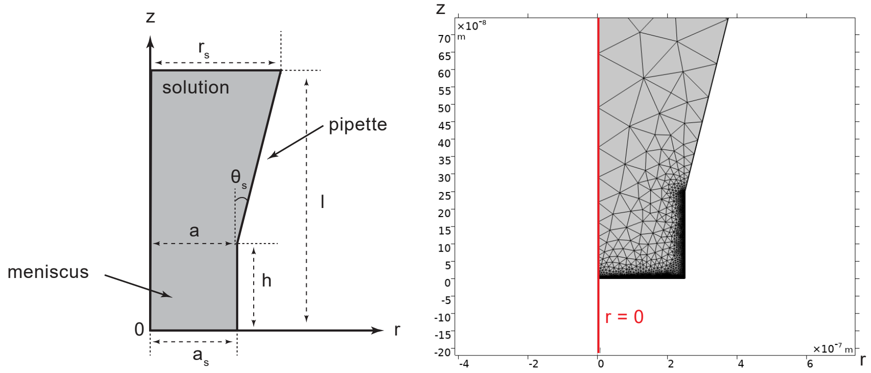

SI Fig. 6: Geometry of the simulation space and an example of mesh used for simulations.

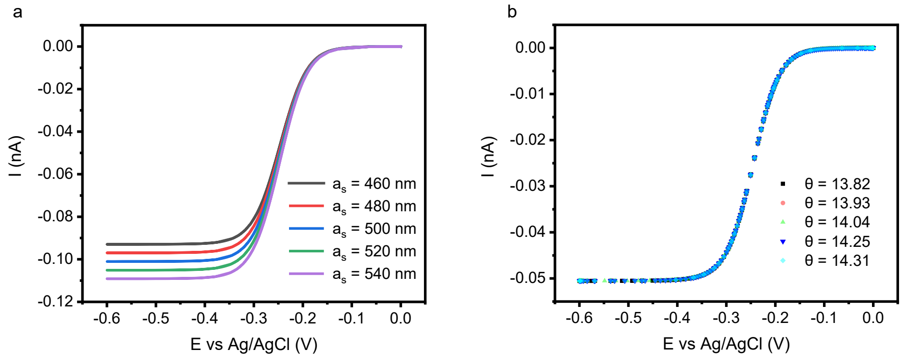

SI Fig. 7: Cyclic voltammogram simulation of 1 mM $Ru(NH_3)_6^{3+}$ in 0.1 M KCl solution obtained as a function of (**a**) the nanopipette radius (a_s), and (**b**) the tangent of the taper angle ($tan\theta$), assuming an electrochemically reversible condition (k_O ¿¿ 1 cm/s).

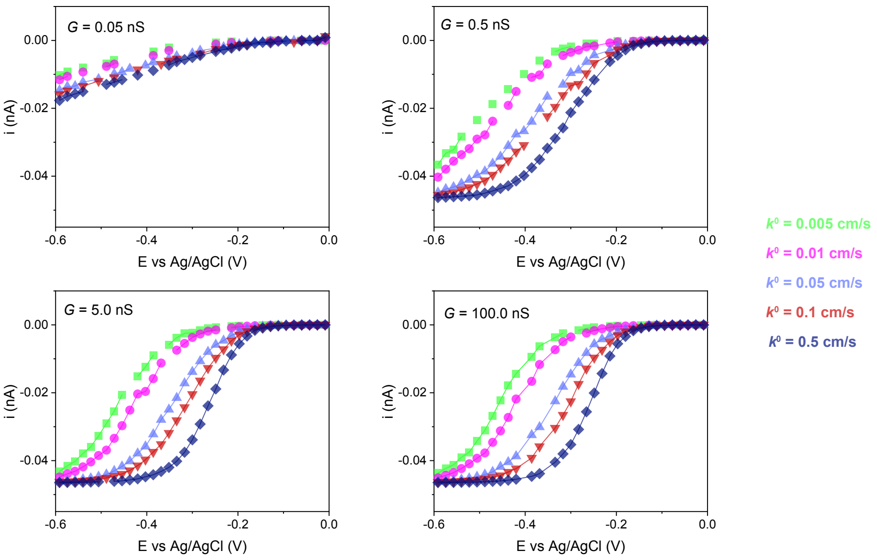

SI Fig. 8: Simulated Cyclic voltammograms of (**a**), 1 mM $Ru(NH_3)_6^{3+}$ in 0.1 M KCl solution on monolayer MoS_2 at different S and k_O values. Scan rate = 200 mV/s

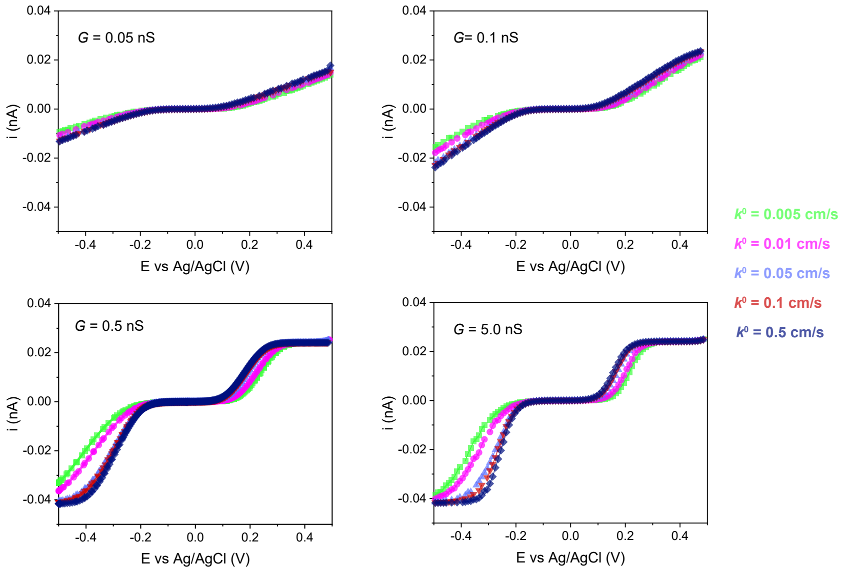

SI Fig. 9: Simulated Cyclic voltammograms of (**a**), 2 mM $Ru(NH_3)_6^{3+}$ and 1 mM FcMeOH in 0.1 M KCl solution on monolayer MoS_2 at different S and k_O values. Scan rate = 200 mV/s

References

(1) Li, H.; Wu, J.; Huang, X.; Lu, G.; Yang, J.; Lu, X.; Xiong, Q.; Zhang, H. Rapid and Reliable Thickness Identification of Two-Dimensional Nanosheets Using Optical Microscopy. *ACS Nano.* **2013**, *7(11)*, 10344–10353.

(2) Zhang, H.; Ma, Y.; Wan, Y.; Rong, X.; Xie, Z.; Wang, W.; Dai, L. Measuring the Refractive Index of Highly Crystalline Monolayer MoS2 with High Confidence. *Scientific Reports* **2015**, *5*, 8440.

(3) Wang, L.; Meric, I.; Huang, P. Y.; Gao, Q.; Gao, Y.; Tran, H.; Taniguchi, T.; Watanabe, K.; Campos, L. M.; Muller, D. A.; Guo, J.; Kim, P.; Hone, J.; Shepard, K. L.; Dean, C. R. One-Dimensional Electrical Contact to a Two-Dimensional Material. *Science* **2013**, *342*, 614–617.

(4) Yu, Y.; Zhang, K.; Parks, H.; Babar, M.; Carr, S.; Craig, I. M.; Winkle, M. V.; Lyssenko, A.; Taniguchi, T.; Watanabe, K.; Viswanathan, V.; Bediako, D. K. Tunable angle-dependent electrochemistry at twisted bilayer graphene with moiré flat bands. *Nat. Chem* **2022**, *14*, 267–273.

(5) Zhan, Y.; Liu, Z.; Najmaei, S.; Ajayan, P.; Lou, J. Large Area Vapor Phase Growth and Characterization of MoS2 Atomic Layers on SiO2 Substrate. *Small (Weinheim an der Bergstrasse, Germany)* **2012**, *8*, 966–71.

(6) Mccreary, K.; Hanbicki, A.; Sivaram, S.; Jonker, B. A- and B-Exciton Photoluminescence Intensity Ratio as a Measure of Sample Quality for Transition Metal Dichalcogenide Monolayers. *APL Materials* **2018**, *6*, 111106.

(7) Edelberg, D. et al. Approaching the Intrinsic Limit in Transition Metal Diselenides via Point Defect Control. *Nano Letters* **2019**, *19*, 4371–4379.

(8) Ebejer, N.; Güell, A. G.; Lai, S. C.; McKelvey, K.; Snowden, M. E.; Unwin, P. R. Scanning Electrochemical Cell Microscopy: A Versatile Technique for Nanoscale Electrochemistry and Functional Imaging. *Annual Review of Analytical Chemistry* **2013**, *6*, 329–351.

(9) Aaronson, B. D. B.; Chen, C.-H.; Li, H.; Koper, M. T. M.; Lai, S. C. S.; Unwin, P. R. Pseudo-Single-Crystal Electrochemistry on Polycrystalline Electrodes: Visualizing Activity at Grains and Grain Boundaries on Platinum for the Fe2+/Fe3+ Redox Reaction. *Journal of the American Chemical Society* **2013**, *135*, 3873–3880.

(10) COMSOL Multiphysics Electrochemistry Module Users Guide, version 5.3, 2017.

(11) Kozbial, A.; Gong, X.; Liu, H.; Li, L. Understanding the Intrinsic Water Wettability of Molybdenum Disulfide (MoS2). *Langmuir* **2015**, *31*, PMID: 26172421, 8429–8435.

(12) Sun, P.; Mirkin, M. V. Kinetics of Electron-Transfer Reactions at Nanoelectrodes. *Anal. Chem* **2006**, *78*, 6526–6534.

(13) Velický, M.; Bradley, D. F.; Cooper, A. J.; Hill, E. W.; Kinloch, I. A.; Mishchenko, A.; Novoselov, K. S.; Patten, H. V.; Toth, P. S.; Valota, A. T.; Worrall, S. D.; Dryfe, R. A. W. Electron Transfer Kinetics on Mono- and Multilayer Graphene. *ACS Nano.* **2014**, *8*, 10089–10100.

(14) Wang, Y.; Kim, C.-H.; Yoo, Y.; Johns, J. E.; Frisbie, C. D. Field Effect Modulation of Heterogeneous Charge Transfer Kinetics at Back-Gated Two-Dimensional MoS_2 Electrodes. *Nano Lett.* **2017**, *17*, 7586–7592.
